# Labeled Bovine Serum Albumin as a Fluorescent Biosensor to Monitor the Stability of Lipid-Based Formulations

**DOI:** 10.3390/bios15070425

**Published:** 2025-07-03

**Authors:** Stefania Bova, Serena Faggiano, Omar De Bei, Marialaura Marchetti, Stefano Bruno, Barbara Campanini, Stefano Bettati, Luca Ronda

**Affiliations:** 1 SITEIA.PARMA, University of Parma, 43124 Parma, Italy; stefania.bova@unipr.it (S.B.); stefano.bettati@unipr.it (S.B.); 2 Department of Food and Drug, University of Parma, 43124 Parma, Italy; serena.faggiano@unipr.it (S.F.); stefano.bruno@unipr.it (S.B.); barbara.campanini@unipr.it (B.C.); 3 Institute of Biophysics, National Research Council, 56124 Pisa, Italy; 4 Department of Medicine and Surgery, University of Parma, 43125 Parma, Italy; omar.debei@unipr.it (O.D.B.); marialaura.marchetti@unipr.it (M.M.)

**Keywords:** albumin, protein biosensor, fluorescence, FRET, acrylodan, lipid-based nanoparticles, phospholipid hydrolysis

## Abstract

In the pharmaceutical field, lipid-based nanoparticles are extensively used for drug or vaccine delivery, particularly for treating respiratory disorders. However, their physico-chemical instability, particularly associated with lipid degradation through hydrolysis or oxidation, can affect their encapsulation properties. To monitor the stability of lipid-based formulations over time, we prepared acrylodan-labeled bovine serum albumin (here called albuminodan), and showed it is a fluorescent biosensor capable of concomitantly detect phospholipids as well as their degradation products, i.e., fatty acids and lysophospholipids. We demonstrated that this tool can be used to follow the distribution of lipids in an aqueous phase and hence could be suitable to characterize the hydrolysis of phospholipids in a lipid-based formulation to monitor the stability of nanoparticles.

## 1. Introduction

Lipid-based nanoparticles, such as liposomes or lipid nanoparticles (LNPs), find central applications in the pharmaceutical field, including drug loading and delivery, and in gene therapy and vaccine formulations, as demonstrated for anti-SARS-CoV2 mRNA delivery [1,2]. Surfactants used for the rescue treatment of respiratory distress syndrome in premature infants (Curosurf^®^) also have a liposomal structure [3].

LNPs consist in spherical assemblies of lipids encapsulating an active substance. The lipids employed in the formulation have generally an amphiphilic nature, carrying a polar head and apolar tails. The currently FDA-approved LNPs are composed of four different lipids, i.e., an ionizable cationic lipid, helper lipids as 1,2-distearoyl-sn-glycero-3-phosphocholine (DSPC), cholesterol, and a polyethylene glycol (PEG)–lipid conjugate. The structural properties of LNPs depend on the ratios of the different components, allowing for different applications [4,5].

While lipid-based nanoparticles offer great advantages over other drug-carrying strategies and widen the possibility of therapeutic intervention, they also bring new analytical challenges, as their chemical and structural complexity requires a careful characterization that differs from that typically used for conventional drugs, either small molecules or biologics. The variety of size, composition, charge, and physico-chemical properties, together with the main reactions causing lipids decomposition, i.e., hydrolysis of ester and/or lipid oxidation, require the development of a robust analytical platform for their characterization and the evaluation of their stability [6]. The rate of degradation processes, for instance, can be affected by parameters such as solution composition and pH, as well as the storage temperature.

The relevance of identifying a standard procedure to assess the integrity of lipids in the development of liposome or LNP drug products is stressed by the specific FDA guidelines [7]. Among the relevant parameters, the physico-chemical stability of the product is listed, and the need for stress and accelerated stability tests is highlighted. The separation and quantification of the original lipids and their corresponding degradation products, i.e., fatty acids (FAs) and lysophospholipids (LPLs), is commonly carried out by coupling chromatographic techniques (reverse-phase HPLC) with different detection systems, such as UV absorption [8], evaporative light scattering detection (ELSD) [9,10], charged aerosol detection (CAD) [11,12], or mass spectrometry [13]. All these methods require a complex development of the procedure and expensive instruments [14]. Simpler colorimetric [15] and fluorometric assays [16], while having a high sensitivity and being suitable for high-throughput screening, cannot distinguish between different lipid species.

Besides these conventional analytical techniques, optical or protein-based systems [17,18,19,20] are an option for quantifying lipid-related molecules. For instance, an intestinal fatty acid-binding protein (iFABP) has been covalently conjugated to a fluorescent probe, 1-(6-(dimethylamino)-2-naphthalenyl)-2-propen-1-one (acrylodan), which binds to the protein fatty acid-binding site. This results in a free fatty acid (FFA) biosensor called ADIFAB (acrylodan-labeled intestinal fatty acid-binding protein) (FFA Sciences) [21,22]. Acrylodan is strongly solvatochromic, i.e., it changes its fluorescence emission wavelength and intensity depending on environmental polarity. When conjugated to iFABP, acrylodan binds to the protein fatty acid-binding site and can be displaced by ligands. The change in solvation environment from the binding site to the aqueous solution causes a fluorescence emission shift used to quantify FFAs. This bioconjugate is commercially available as a probe to determine FFAs concentration in aqueous solutions, binding affinities of fatty acid-binding proteins, membrane/water partition coefficients, lipase activities, and serum levels of FFAs [21]. More recently, it has been used to characterize the hydrolysis of phospholipids (PLs) from a synthetic pulmonary surfactant, CHF5633, to evaluate its stability [23,24]. While this study, as well as another work where PL hydrolysis was enzymatically promoted [25], demonstrated the capability of ADIFAB to monitor liposome degradation, this sensor was unable to discriminate between different ligands.

In this work, we take a step forward in the setup of a protein-based tool to characterize lipid-based nanoparticles by developing a new bioconjugate. The analysis of spectral changes in this biosensor is directed towards the possibility of simultaneous detection of PLs and their hydrolysis products. We have coupled the capability of bovine serum albumin (BSA), a cheap and easily accessible protein, to bind multiple copies of long-chain fatty acids [26,27] with the fluorescence properties of acrylodan, conjugated to the only reactive cysteine (Cys34) of the protein. We monitored the complete fluorescence spectra (both Trp and acrylodan emission) in the presence of different lipid ligands at different concentrations, demonstrating they exhibit similar affinities but different fluorescence spectral features. These reference titrations have been used to follow the distribution of lipid species in the aqueous phase in a lipid-based formulation under accelerated stability tests. We thus demonstrated the capability of acrylodan-conjugated BSA (hereafter called albuminodan) to detect hydrolysis products occurring in a formulation containing lipids.

## 2. Materials and Methods

### 2.1. Reagents

All chemicals were of highest available purity and were used as received without further purification. Acrylodan was provided by Molecular Probes™ (Eugene, OR, USA) and was solubilized in 100% dimethyl sulfoxide (DMSO) at 10 mg/mL. BSA, oleic and palmitic acid and phospholipase A_2_ (PLA_2_) were purchased by Sigma-Aldrich (St. Louis, MO, USA). Dipalmitoyl phosphatidylcholine (DPPC), 1-palmitoyl-2-oleoyl-sn-glycero-3-(phospho-rac-(1-glycerol)) (POPG), and lysopalmitoylphosphatidylcholine (LPPC)—as mixture of 1-LPPC and 2-LPPC—were provided by CordenPharma (Basel, Switzerland). Lysopalmitoylphosphatidylglycerol (LPPG) and lysooleilphosphatidylglycerol (LOPG) were provided by Avanti Research (Alabaster, AL, USA).

### 2.2. Bovine Serum Albumin Conjugation

BSA conjugation with acrylodan was carried out using a modified version of Wang’s protocol [28]. Briefly, a 50 µM solution of BSA in 100 mM phosphate buffer, pH 7.0, was mixed with an acrylodan solution in DMSO to reach a 1:1 molar ratio. The final DMSO concentration was kept below 2% to avoid any solvent effect on the protein. The reaction mixture was maintained for 1 h at 20 ± 0.5 °C in a thermostatted bath, and then unreacted acrylodan was removed via diafiltration on cellulose-regenerated 10 kDa centrifugal devices (Merck-Millipore, Darmstadt, Germany). The absorption spectrum was then recorded using a Cary 4000 UV–Vis spectrophotometer (Agilent, Santa Clara, CA, USA) to estimate the protein (280 nm) and probe (384 nm) concentrations and the degree of labeling (DOL), i.e., the ratio of probe concentration to total protein concentration.

### 2.3. Fluorescence Measurements on Acrylodan-Conjugated BSA (Albuminodan)

Fluorescence emission spectra of albuminodan upon excitation at 280 and 384 nm in the absence and presence of ligands were collected with a Fluoromax^®^-3 spectrofluorometer (Horiba Jobin Yvon, Tokyo, Japan), equipped with a thermostatted bath kept at a constant temperature of 20 ± 0.5 °C. A 3 × 3 mm quartz microcuvette was used. Slits were set at 3.5 nm and the integration time to 0.3 s.

Standard stock solutions of PLs, LPLs, and FAs were prepared by solubilizing the reagents in 100% ethanol to 500 µM concentration. Serial dilutions with PBS were carried out to keep ethanol concentration below 25% of the final mixture. For fluorescence titration of pure species, 69 µL of a solution containing 500 nM of albuminodan in PBS buffer was titrated with pure species to cover the 0.01–9.2 µM concentration range. Spectra were collected upon 2 min of incubation. For stoichiometric titrations, different concentrations were used: 1.5 µM for albuminodan and from 1 to 24 µM for ligands. Their limited solubility prevented them from being tested in a large excess, which could affect the accuracy of determining the intersection point and, consequently, the stoichiometric ratio.

Kinetic experiments to monitor the spontaneous hydrolysis of a lipid-based formulation were carried out by adding 1 µL of the formulation to a 69 µL volume of 500 nM albuminodan.

### 2.4. Preparation of DPPC/POPG Formulation

A lipid-based test formulation was obtained via sonication using a modified version of Morrisey’s protocol for the generation of phospholipid small unilamellar vesicles (SUVs) [29]. Initially, 1 mL each of two synthetic PL stock solutions—DPPC and POPG—at 1 mM concentration in ethanol were mixed in a 1:1 molar ratio, for a total amount of 2.6 µmoles of phospholipids. The mixture was subsequently dried using an RVC 2-18 SpeedDry Vacuum concentrator (Martin Christ, Osterode am Harz, Germany). The dried PLs were rehydrated with 2.6 mL (482 µM of PLs as final concentration) of an aqueous solution containing 20 mM HEPES, 100 mM NaCl, and pH 7.5, followed by incubation at room temperature for 1 h. Vortexing the suspension produced a milky dispersion, which was then subjected to sonication for 30 min until the solution became nearly transparent. The final DPPC/POPG formulation was left to equilibrate O/N at 4 °C before starting stability evaluations. The formulation hydrolysis was then followed leaving the formulation at the same temperature.

### 2.5. PLA_2_ Hydrolysis Experiment

PLA_2_ was employed to catalyze the hydrolysis of PLs of the formulation and perform an accelerated hydrolysis test. The enzyme was reconstituted at 1 mg/mL concentration in 25 mM Tris, 140 mM NaCl, and pH 7.4 and incubated with the formulation at a final concentration of 0.014 mg/mL (1 µM). Hydrolysis was conducted in a buffer composed of 25 mM Tris and 1 mM CaCl_2_, pH 8.0 at 37 °C. The reaction was monitored by sampling a fixed volume of formulation (1 µL) over time and collecting fluorescence spectra under the same experimental conditions used for the standard ligands.

### 2.6. Spectral Analysis

To investigate how the fluorescence signal of albuminodan changes with increasing concentration of pure species, we first analyzed the spectral data using singular value decomposition (SVD). This mathematical method simplifies complex datasets by extracting the main patterns of variation, helping to distinguish the contributions of different fluorescent species or intermediates. To estimate the uncertainty associated with SVD, jackknife resampling (leave-one-out variant) was applied.

The sensing properties of albuminodan were tested by adding lipid-based formulations and collecting fluorescence spectra at different time points. Each of these spectra was assumed to be a mixture of fluorescent species corresponding to pure species. To estimate the spectral contribution of each species to the experimental signal, a linear spectral deconvolution was performed. Each experimental spectrum was represented as a weighted sum of the spectra of the pure species. The weights, corresponding to the contribution of each species, were optimized using MATLAB’s FMINCON function, which solves constrained nonlinear optimization problems. This algorithm adjusted the coefficients to minimize the difference between the measured spectrum and the reconstructed one. These weights were subsequently converted into percentages relative to the total available albuminodan.

The spectral analysis was carried out with MATLAB R2024b [30].

Dissociation constants (*K_D_*) of the complex of albuminodan with pure ligand species (*P:L*) were determined by fitting the first eigenvalue from SVD as a function of ligand concentration to a tight binding isotherm (Equation (1)):

(1) $$\left[P:L\right]=\frac{\left(\left[P\right]+\left[L\right]+K_{D})-\sqrt{\left([P\right]+\left[L\right]+K_{D})-4[P][L}\right]}{2}$$

where *P* is ligand-free albuminodan, *L* is ligand, and *K_D_* is dissociation constant.

## 3. Results and Discussion

### 3.1. Structural Comparison Between Human and Bovine Serum Albumin

**Figure 1:**
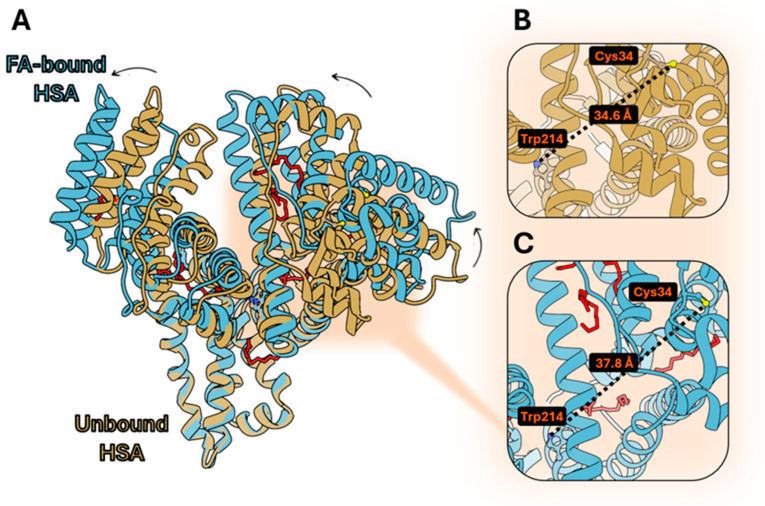
(**A**) Superimposition of ligand-free HSA (PDB: 1AO6), and FA-bound HSA (PDB: 8RCP); (**B**) distance calculated between Cys34 and Trp214 on ligand-free HSA; (**C**) distance calculated between Cys34 and Trp214 on FA-bound HSA. The UCSF Chimera 1.18 software was used.

Starting from the observation that serum albumins are known for their ability to bind several lipophilic molecules, we investigated the structural features and the conformational changes induced by ligand binding in the most commonly available ones, i.e., human serum albumin (HSA) and bovine serum albumin (BSA). The binding of FAs to HSA has been characterized from a structural point of view. In particular, the conformation rearrangements occurring upon myristate [31,32] (8RCP) or oleate [33] binding were characterized, showing substantial domain rotations, FA binding cavity expansion, and the rotation of hydrophobic side chains gating the binding sites (Figure 1A). The possibility to homogeneously conjugate HSA on the uniquely reacting cysteine (Cys34) with acrylodan has already been explored, and the fluorescence properties of the bioconjugate have been reported [33]. The distance between Trp214 and the labeled cysteine in ligand-free HSA is 34.6 Å (Figure 1B), in principle allowing for FRET experiments [34]. Conformational changes occurring upon pH variation modulate this distance [35]. Also, FA binding causes conformational changes in conjugated HSA, with an enhancement of the solvent exposure of acrylodan, as demonstrated by acrylamide quenching experiments [33]. This evidence demonstrated that the conformational rearrangements occurring in HSA when FAs bind are sensed as a modification in the relative position of Trp214 and Cys34, which distance resulted to be 37.8 Å (Figure 1C), and FRET could be exploited as a measure of these ligand-induced changes.

**Figure 2:**
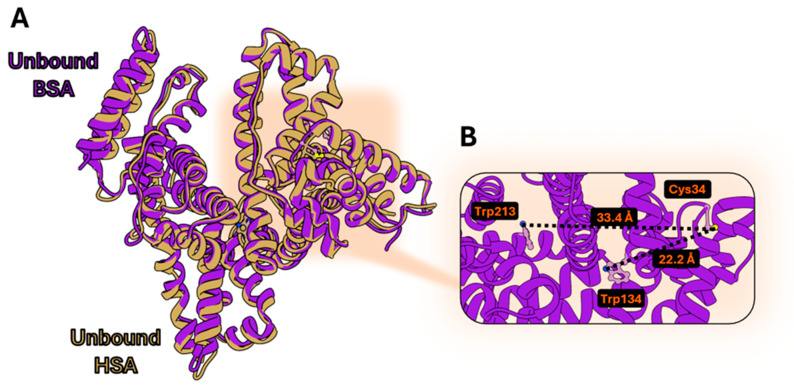
(**A**) Superimposition of ligand-free HSA (PDB: 1AO6), and BSA (PDB: 4OR0); (**B**) distances calculated between ligand-free BSA Cys34 and Trp213 or Trp134. The UCSF Chimera 1.18 software was used.

BSA, while sharing a high structural similarity with HSA (Figure 2A), has an additional Trp residue (Trp134) whose distance to Cys34 is 10 Å lower than that calculated for Trp213 (corresponding to Trp214 in HSA) (Figure 2B). Trp134 could significantly contribute to a higher FRET efficiency due to a shorter distance to the site of fluorophore conjugation and hence offer a second sensing element towards the conformational variations originated by ligand binding.

Based on these structural evaluations, BSA was chosen as a scaffold for acrylodan conjugation to produce albuminodan to bind and detect different lipids in the aqueous phase of lipid-based nanoparticle formulations. The sensing principle differs from the commercially available fatty acid fluorescence sensor ADIFAB, in which the fluorophore is buried inside the fatty acid-binding site. Ligand binding to ADIFAB causes fluorophore displacement, generating signal changes independent on the chemical nature of the ligand [36]. In albuminodan, the fluorophore is not expected to be displaced by FAs, since they bind to different pockets. Therefore, albuminodan could sense, through emission intensity variation and solvatochromic changes in both Trp residues and acrylodan, ligand-dependent conformational changes. Based on this hypothesis, we envisioned that albuminodan can be a ligand-discriminating (i.e., PLs, LPLs, and FAs) protein biosensor.

### 3.2. BSA Spectroscopic Evaluation

Before conjugation, we collected Trp fluorescence spectra on BSA upon excitation at 280 nm. The emission peaks were centered at 347 nm, differently from HSA, where only Trp214 is present, for which emission is reported around 335 nm [37]. The red-shifted emission of the bovine protein with respect to the human homolog is consistent with the presence in the former of a second Trp (Trp134) located in a hydrophilic environment close to the protein surface. Based on the structure, the other fluorescent residue, Trp213, is surrounded by hydrophobic residues as Trp214 in HSA.

### 3.3. BSA Conjugation to Acrylodan

**Figure 3:**
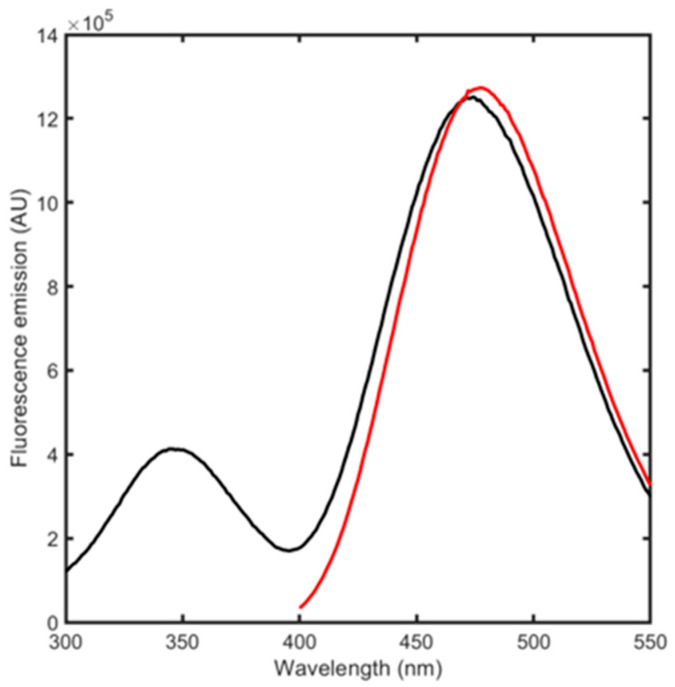
Fluorescence emission spectra upon excitation at 384 nm (red) and at 280 nm (black) of 500 nM albuminodan.

BSA conjugation with acrylodan was carried out by adapting an existing protocol [28]. The reaction occurred with a high yield, producing a degree of labeling (DOL) of ~1, indicating that, as expected, one acrylodan molecule is bound for each protein molecule (Figure S1). The fluorescence emission spectra were then acquired, directly exciting acrylodan at 384 or 280 nm (Figure 3). This latter condition can produce both Trp and acrylodan excitation as well as acrylodan emission through energy transfer from Trp. Acrylodan excitation resulted in a fluorescence peak centered at 478 nm while 280 nm excitation generated the fluorescence emission of both Trp residues and of acrylodan for energy transfer, with peaks centered at 347 and 470 nm. We proceeded by using this signal to monitor ligand binding and its related conformational changes, since the complete emission spectra allows for obtaining the highest possible variability upon binding between different ligands.

### 3.4. Albuminodan Binding to PLs, LPLs, and FAs

**Figure 4:**
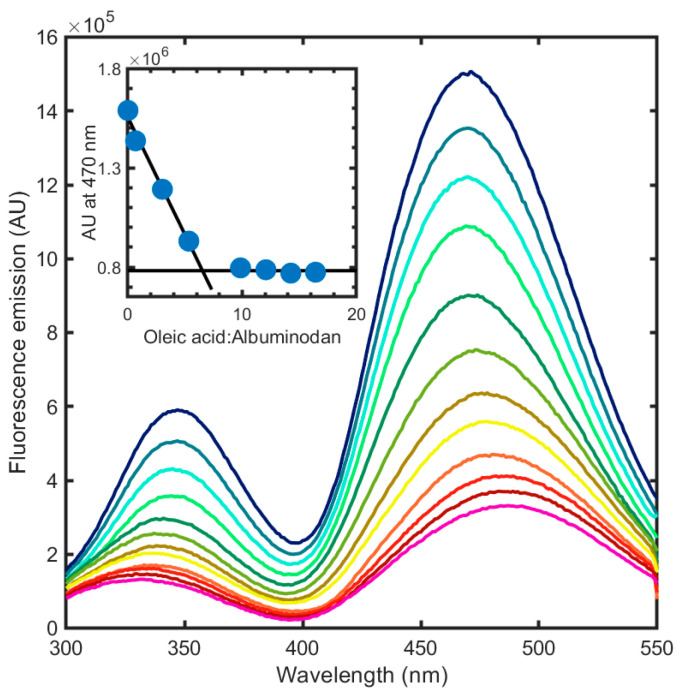
Fluorescence emission spectra upon excitation at 280 nm of albuminodan (blue line) in the presence of increasing concentrations of oleic acid, ranging from 0.01 to 9.2 µM (from blue to pink line). Inset: stoichiometric binding reported as fluorescence intensity at 470 nm as a function of the oleic acid:albuminodan molar ratio. The calculated ratio is equal to 6.

Once we determined the spectral properties of albuminodan, we measured fluorescence spectra in the presence of increasing concentrations of different ligands: two synthetic phospholipids, DPPC and POPG, and their corresponding hydrolysis products, i.e., 1-LPPC, 2-LPPC, LPPG, and LOPG as LPLs, and oleic acid and palmitic acid as FAs. As a first step, we aimed to determine if this bioconjugate was able to sense ligand binding by changing its fluorescence emission and was amenable to evaluate binding stoichiometry. Starting from FAs, the simplest structural unit of phospholipids, for which BSA is reported to have six binding sites [26], we measured the binding of oleic acid to albuminodan by adding increasing amounts of ligand to a 500 nM albuminodan solution and recording the corresponding emission spectra upon excitation at 280 nm (Figure 4). A large emission decrease was observed on both direct Trp emission and acrylodan emission. Emission peaks for both Trp and acrylodan shifted at increasing ligand concentration. The binding of 9.2 µM oleic acid caused a ~80% emission decrease for both Trp and acrylodan, together with a hypsochromic shift for the former (from 347 nm to 332 nm) and a bathochromic shift for the latter (from 470 nm to 490 nm). As a control experiment, direct acrylodan excitation gave a slightly different peak shift and a fluorescence decrease comparable to that obtained by 280 nm excitation. These spectral changes likely reflect the environmental changes for the intrinsic and extrinsic fluorophores and the conformational changes caused by ligand binding. As expected, within the limit of the analysis (see Section 2.3) the stoichiometry for oleic acid:albuminodan complex reached 6:1, since spectra did not significantly change over this ratio (Figure 4, inset), in agreement with literature for unmodified BSA [26], demonstrating that protein chemical modification did not impair BSA ligand-binding capacity. As a comparison, acrylodan-labeled HSA showed a bathochromic shift from 476 nm to 483 nm and a 20% emission decrease upon binding oleic acid in a 5:1 molar ratio [33], suggesting that albuminodan is more sensitive in terms of fluorescence changes with respect to the corresponding HSA conjugate.

**Figure 5:**
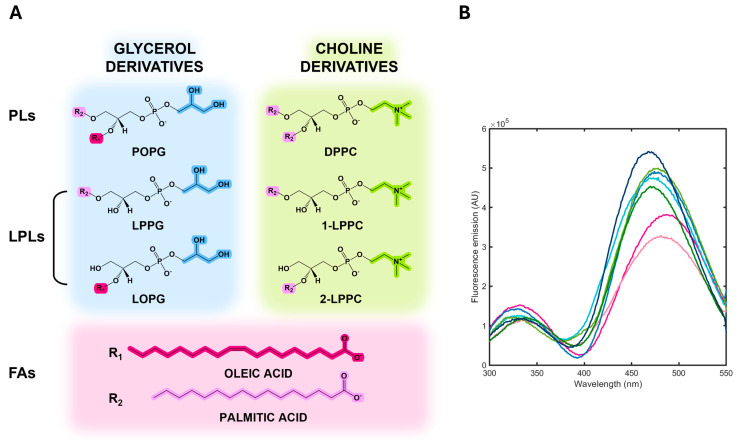
(**A**) Chemical structure of PLs, LPLs, and FAs tested. (**B**) Fluorescence emission spectra upon excitation at 280 nm of albuminodan with saturating concentrations of DPPC (dark green), POPG (dark blue), LPPG (light blue), LOPG (cyan), LPPC (light green), oleic acid (magenta), and palmitic acid (light pink).

Moreover, as a further test to evaluate albuminodan performance, we collected emission spectra on unmodified BSA upon 280 nm excitation in the absence and presence of 9.2 µM oleic acid. By comparing these data with those in Figure 4, we observed a 58% decrease in the signal at 347 nm, compared to an 80% decrease in acrylodan-labeled albumin at the same wavelength (Figure S2). This suggests that the presence of conjugated acrylodan induces a larger variation in the tryptophan signal than BSA alone. After determining the binding competence of albuminodan for oleic acid, we tested all other ligands (Figure 5A). PLs, LPLs, and FAs bound albuminodan with the same stoichiometry. However, the fluorescence spectra at saturating conditions were different (Figure 5B). Therefore, we envisioned that signal variations could depend on the nature of the lipid ligand. The resulting spectra for albuminodan in the presence of a mixture of different lipids would thus reflect the distribution of ligands free in solution, for which quantification can be obtained through spectral deconvolution.

We carried out titrations to determine the affinity of albuminodan for PLs, LPLs, and FAs. As expected, based on spectra at ligand-saturating conditions, the fluorescence spectra showed complex spectral changes, with variation in terms of emission intensity and Stokes shift for both the Trp and acrylodan peaks (Figure S3). Since BSA has six binding sites, we carried out a singular value decomposition (SVD) on data matrix from all titrations to possibly identify and characterize different binding sites having different affinities and giving different spectral changes.

**Figure 6:**
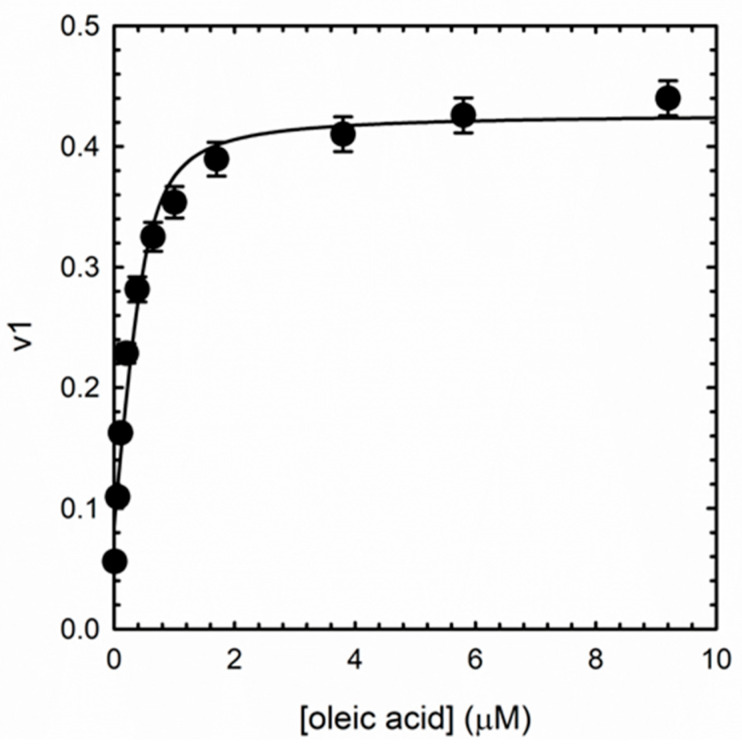
First component weight (eigenvalue) as a function of oleic acid concentration. The line through data points represents fitting to Equation (1).

For all ligands, SVD analysis resulted in two meaningful components, accounting for 99.8% and 0.2% of the variance for the first and second component, respectively. We hypothesized that the first component represents ligands binding to the protein and coupled large conformational events, while the second component could be related to residual uncoupled conformational events caused by ligand binding (Figure S4). The largely predominant presence of a single component supports a binding mechanism in which ligand binding to different sites on the protein causes the same spectral effect. We analyzed the weight (eigenvalue) of the first SVD component (v1) as a function of concentration for the different ligands to determine the binding affinity (the curve for oleic acid is reported in Figure 6) using a tight binding equation (Equation (1)) which considers a 6:1 ligand:albuminodan ratio. The equation gave a good fit for all ligands, without the need to introduce more than one binding species, demonstrating that, for each lipid, all binding sites also share an overlapping affinity. The determined dissociation constants are reported in Table 1.

Dissociation constants were quite similar for all ligands, ranging from ~0.1 to 0.4 µM (Table 1 and Figure S5), with no affinity grouping with respect to the chemical category (PLs vs. LPLs or FAs).

### 3.5. Monitoring the Hydrolysis of a DPPC/POPG Formulation Induced by Phospholipase A_2_ (PLA_2_)

Since phospholipids such as DPPC and POPG are the main constituents of lipid-based nanocarriers, we tested albuminodan as an easy-to-use tool to check the chemical stability of lipid-based nanoparticle formulations by detecting the presence of different molecules (PLs and their hydrolysis products) free in solution as a consequence of PLs degradation.

We prepared liposomes composed of an equal amount of DPPC and POPG as a model of synthetic PLs used for liposome formulations. Details on the preparation of the formulation are reported in the Materials and Methods section.

Firstly, we added increasing amounts of this formulation to albuminodan to confirm the capacity of the biosensor to bind a fraction of the formulation components free in solution. Adding increasing amounts of the DPPC/POPG formulation to albuminodan resulted in a linear change in the spectra with each subsequent addition (Figure S6). These results confirmed the capacity of albuminodan to bind lipid-based formulation components.

We therefore checked the ability of this probe to monitor possible spontaneous hydrolysis reactions that could take place in the formulation over time. Moreover, as an accelerated hydrolysis test, we also added to the formulation the enzyme PLA_2_, which catalyzes the hydrolysis at the sn-2 position of phospholipids by cleaving the covalent bond between glycerol and fatty acids. This enzyme is reported to be more active towards glycerophospholipids with unsaturated fatty acids, such as oleic acid [38,39], suggesting a preferential hydrolysis of POPG with respect to DPPC.

**Figure 7:**
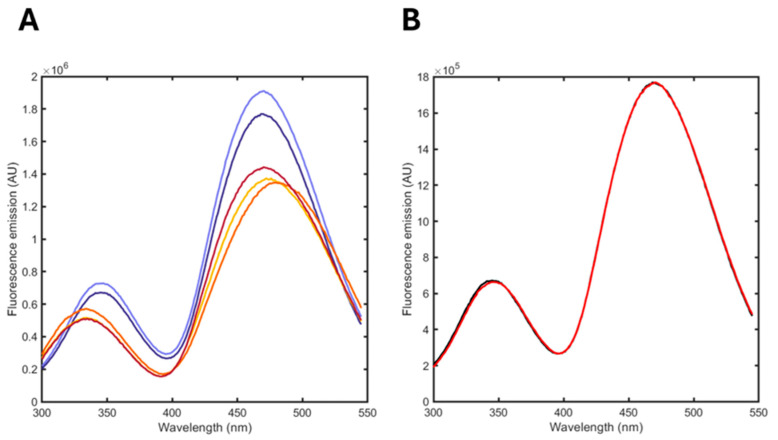
(**A**) Fluorescence emission spectra upon excitation at 280 nm of albuminodan in the presence of DPPC/POPG formulations. Spontaneous hydrolysis: t = 0 (dark violet), 2 months (light violet); enzymatic hydrolysis (PLA_2_): 2 min (yellow), 1 h (orange), 6 h (dark red). (**B**) Comparison between an experimental fluorescence emission spectrum of albuminodan in the presence of DPPC/POPG formulation (black) and the reconstructed spectrum from linear combination of standard spectra (red).

We followed the hydrolysis kinetics of the formulation incubated in the absence and presence of PLA_2_ by recording spectra of albuminodan upon the addition of a fixed volume of formulation at different time points (Figure 7A).

The spectra demonstrated that albuminodan reports a change in both spontaneous and enzymatically promoted hydrolysis, with a much higher effect on the latter, as expected due to the enzymatic action of PLA_2_.

The analysis was performed by fitting the spectra at different time points as a linear combination of the reference spectra acquired at saturating conditions of the single ligands (DPPC, POPG, LPPG, LOPG, LPPC, oleic acid and palmitic acid). Figure 7B reports a representative fit, showing the good agreement between the calculated and observed spectrum. The relative fraction of each component is reported in Table 2.

The analysis reported the pure species released in the aqueous phase that bind albuminodan. The formulation just upon preparation (t 0) showed an equilibrium between unbound BSA and POPG, with no traces of DPPC. This reflects the fact that the former PL has a higher solubility as a monomer in solution due to electrostatic repulsion of the phosphate group. DPPC, having a head group with a zwitterionic nature, has a much lower water solubility and is not easily solvated as a monomer. After two months, the fluorescence spectra remained quite similar, and a fraction of DPPC-bound albuminodan appeared that could originate from a PLs redistribution over time making DPPC more directly accessible to the protein. It emerged from this analysis that the hydrolysis of PLs did not proceed significantly in the formulation over this time window. Low fractions of degradation of phospholipids, marginally detected by albuminodan, should not be relevant for the stability of a formulation.

The fluorescence spectra on formulation incubated in the presence of PLA_2_ already showed a marked change 2 min after the addition, which we found to be related to the appearance of significant fractions of oleic acid and LPPG, as expected based on the selectivity of the enzyme and its catalytic fitness. POPG was still present as well as DPPC, this latter PL possibly made accessible to albuminodan thanks to the SUV destabilization caused by PLA_2_. Analyses of the spectra after 1 and 6 h showed a decrease in POPG as a consequence of the catalyzed reaction of phospholipase, and a concomitant increase in oleic acid or LPPG. These two latter molecules did not show linear kinetics, possibly due to two overlapping phenomena: a rapid enzymatic hydrolysis and a slower SUV destabilization and reorganization. In evaluating the resulting percentages of the analysis, we had to consider that several events can influence the fraction of the single ligand bound: (i) The affinity of albuminodan for different ligands, while similar, thus allowing for the detection of all of them in the same range of concentration, is not exactly the same; hence, there is not a strictly direct correlation between saturation and concentration in solution. (ii) The partition coefficients of ligands between water and SUV/micelles are likely different. (iii) The hydrolysis reaction can impact the partition coefficients since the presence of the degradation products affects membrane properties. The contributions of these factors to the fraction of bound ligands are not comparable; while the albuminodan affinity for ligands ranges in a factor of 3–4, the differences in solubility are much higher, spanning between orders of magnitude, as well as the partition coefficients, which in any case cannot be considered a constant for each molecule but the result of several factors characterizing the specific formulation. For these reasons, the percentages we observed cannot be an exact quantification of PL hydrolysis, but they can be considered a report of the species present in the water phase. Indeed, these values could be a fingerprint to be correlated with other properties of lipid-based nanoparticles such as melting point, loading capacity, and other stability-related parameters, so they can have analytical potential for formulation characterization.

We conclude that albuminodan could be a valuable tool to monitor liposome stability and lipid composition in addition to already assessed methods, as it combines the ease of use of collecting fluorescence spectroscopic data with some (indirect) molecular information that could be obtained by applying more technically demanding and labor-intensive techniques.

## Figures and Tables

**Table 1:** Dissociation constants of albuminodan with ligands.

Ligand	*K*_*D*_ (μM)
DPPC	0.137 ± 0.070
POPG	0.243 ± 0.087
LPPG	0.218 ± 0.079
LOPG	0.187 ± 0.050
LPPC	0.183 ± 0.057
oleic acid	0.104 ± 0.046
palmitic acid	0.395 ± 0.105

**Table 2:** Relative percentage of pure components (best-fit coefficients obtained from *fmincon*) as a function of time during DPPC/POPG formulation spontaneous or enzymatic hydrolysis.

	Spontaneous Hydrolysis (%)		Enzymatic Hydrolysis (%)		
	t = 0	t = 2 mos	t = 2 min	t = 1 h	t = 6 h
ligand-free albuminodan	71	66	4	4	6
oleic acid	0	0	54	83	11
palmitic acid	0	0	0	0	0
LPPG	0	0	4	0	51
LPPC	0	0	0	0	0
LOPG	0	0	0	0	0
DPPC	0	20	18	13	31
POPG	29	14	20	0	1
R^2^	0.9996	0.9996	0.9978	0.9953	0.9969

## Data Availability

Raw data are available upon reasonably request to interested researchers.

## Supplementary Materials

The following supporting information can be downloaded at https://www.mdpi.com/article/10.3390/bios15070425/s1: Figure S1: Absorption spectrum of albuminodan; Figure S2: BSA and albuminodan emission spectra in the presence and absence of oleic acid. Figure S3: Fluorescence emission spectra of albuminodan in presence of PLs, LPLs, and FAs; Figure S4: First and second eigenvectors from SVD on each tested ligand with albuminodan; Figure S5: First component eigenvalue as a function of increasing PL, LPL, and FA concentrations and corresponding fittings; Figure S6: Fluorescence emission spectra of albuminodan upon addition of a DPPC/POPG lipid-based formulation.

## Abbreviations

The following abbreviations are used in this manuscript:

LPNs	Lipid nanoparticles
DSPC	1,2-distearoyl-sn-glycero-3-phosphocholine
PEG	Polyethylene glycol
FAs	Fatty acids
LPLs	Lysophospholipids
ELSD	Evaporative light scattering detection
CAD	Charged aerosol detection
iFABP	Intestinal fatty acid-binding protein
FFA	Free fatty acid
PLs	Phospholipids
BSA	Bovine serum albumin
FRET	Fluorescence resonance energy transfer
DMSO	Dimethyl sulfoxide
PLA_2_	Phospholipase A_2_
DPPC	Dipalmitoyl phosphatidylcholine
POPG	1-palmitoyl-2-oleoyl-sn-glycero-3-(phospho-rac-(1-glycerol))
LPPC	Lysopalmitoylphosphatidylcholin
LOPG	Lysooleilphosphatidylglycerol
LPPG	Lysopalmitoylphosphatidylglycerol
SUV	Small unilamellar vesicles
HSA	Human serum albumin
DOL	Degree of labeling
SVD	Singular value decomposition
