# Supplementary material for: Labeled Bovine Serum Albumin as a Fluorescent Biosensor to Monitor the Stability of Lipid-Based Formulations
Source: Biosensors (Basel). 2025 Jul 3;15(7):425. doi: 10.3390/bios15070425 (PMC12293067; doi:10.3390/bios15070425)
Supplement: Supplementary file 1 [file biosensors-15-00425-s001.zip › biosensors-3702691-supplementary.pdf]

Supplementary Materials

Labeled Bovine Serum Albumin as a Fluorescent Biosensor to Monitor the Stability of Lipid-based Formulations

Stefania Bova [1], Serena Faggiano [2,3], Omar De Bei [4], Marialaura Marchetti [4], Stefano Bruno [2], Barbara Campanini [2], Stefano Bettati [1,3,4] and Luca Ronda [3,4,*]

[1] SITEIA.PARMA, University of Parma, 43124 Parma, Italy; stefania.bova@unipr.it (S.B.); stefano.bettati@unipr.it (S.B.)
[2] Department of Food and Drug, University of Parma, 43124 Parma, Italy; serena.faggiano@unipr.it (S.F.); stefano.bruno@unipr.it (S.B.); barbara.campanini@unipr.it (B.C.)
[3] Institute of Biophysics, National Research Council, 56124 Pisa, Italy
[4] Department of Medicine and Surgery, University of Parma, 43125 Parma, Italy; omar.debei@unipr.it (O.D.B.); marialaura.marchetti@unipr.it (M.M.)
* Correspondence: luca.ronda@unipr.it; Tel.: +39-0521-905502

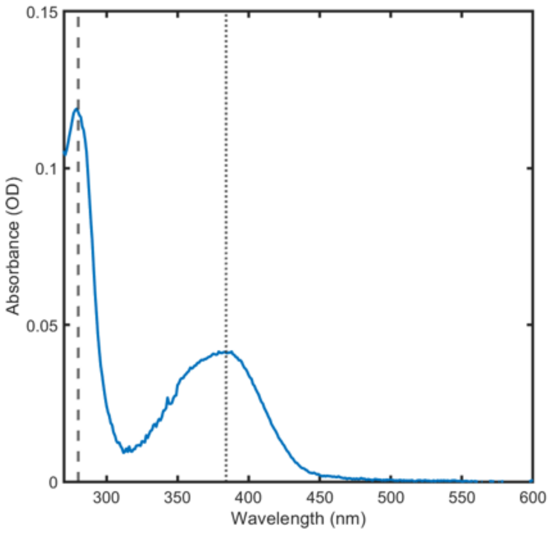

Figure S1. Absorption spectrum of albuminodan with reference blue lines at 280 nm (dashed) and 384 nm (dotted) collected using 1 cm pathlength cuvette. The molar extinction coefficients are 49915 M^{-1} cm^{-1} at 280 nm for BSA and 16075 M^{-1} cm^{-1} at 365 nm and 15868 M^{-1} cm^{-1} at 280 nm for acrylodan.

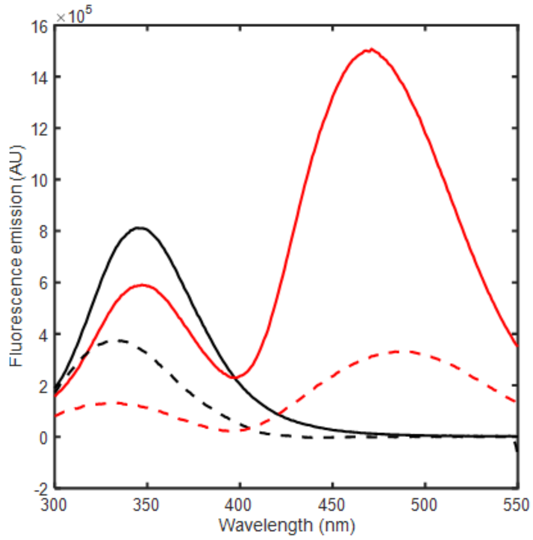

Figure S2. Fluorescence emission spectra of BSA upon 280 nm excitation in the absence (solid black) and in presence (dashed black) of 9.2 μM oleic acid in comparison with the corresponding emission spectra of albuminodan upon 280 nm excitation in the absence (solid red) and in presence (dashed red) of 9.2 μM oleic acid.

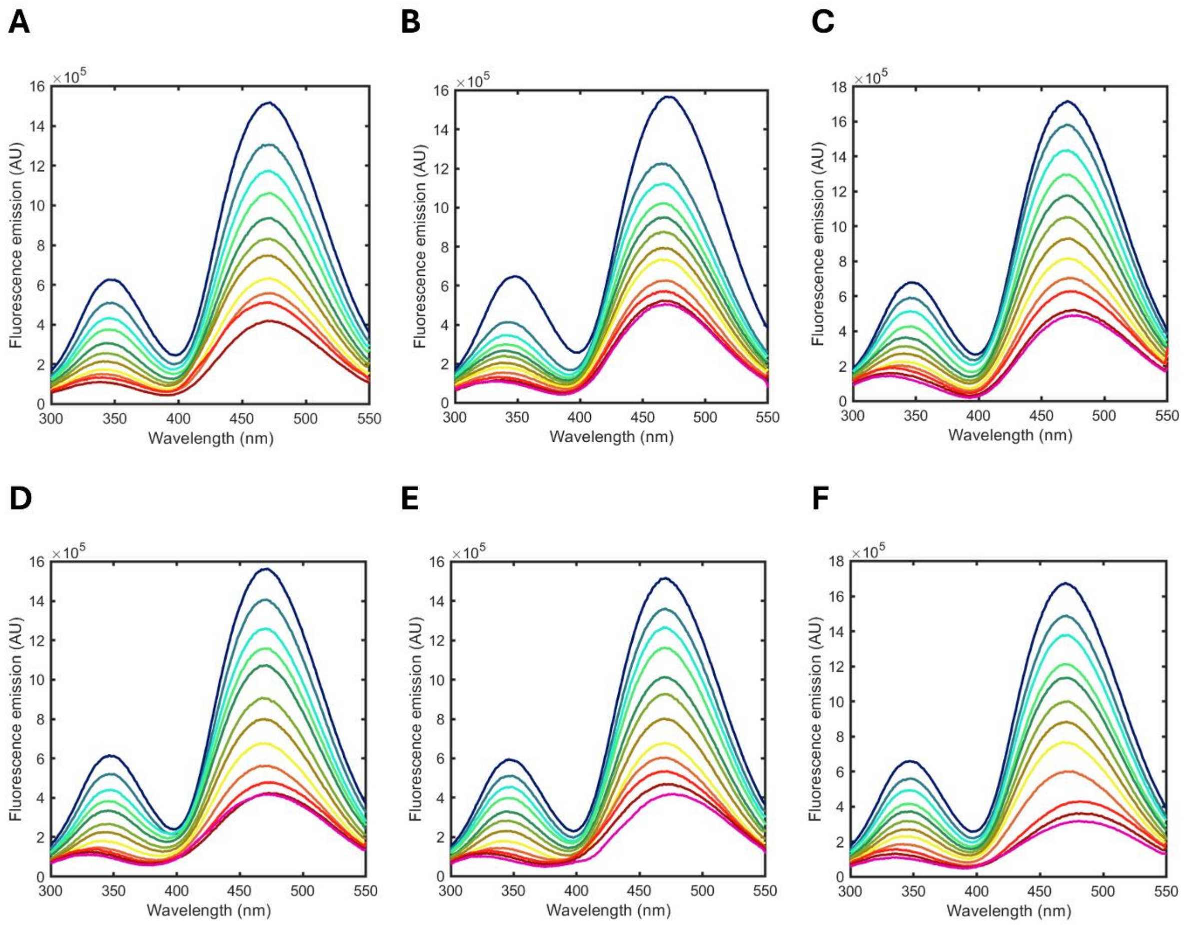

Figure S3. Fluorescence emission spectra upon excitation at 280 nm of albuminodan (dark blue) in presence of 10 nM (light blue), 58 nM (turquoise), 105 nM (bright green), 199 nM (deep green), 383 nM (olive green), 654 nM (dark yellow), 1 µM (bright yellow), 1.7 µM (dark orange), 3.8 µM (bright red), 5.8 µM (dark red), 9.2 µM (pink) of DPPC (A), POPG (B) LPPG (C), LOPG (D), LPPC (E), palmitic acid (F), after two minutes of incubation: 69 µL of a solution containing 500 nM of albuminodan in PBS buffer was titrated with pure species to cover the concentration range.

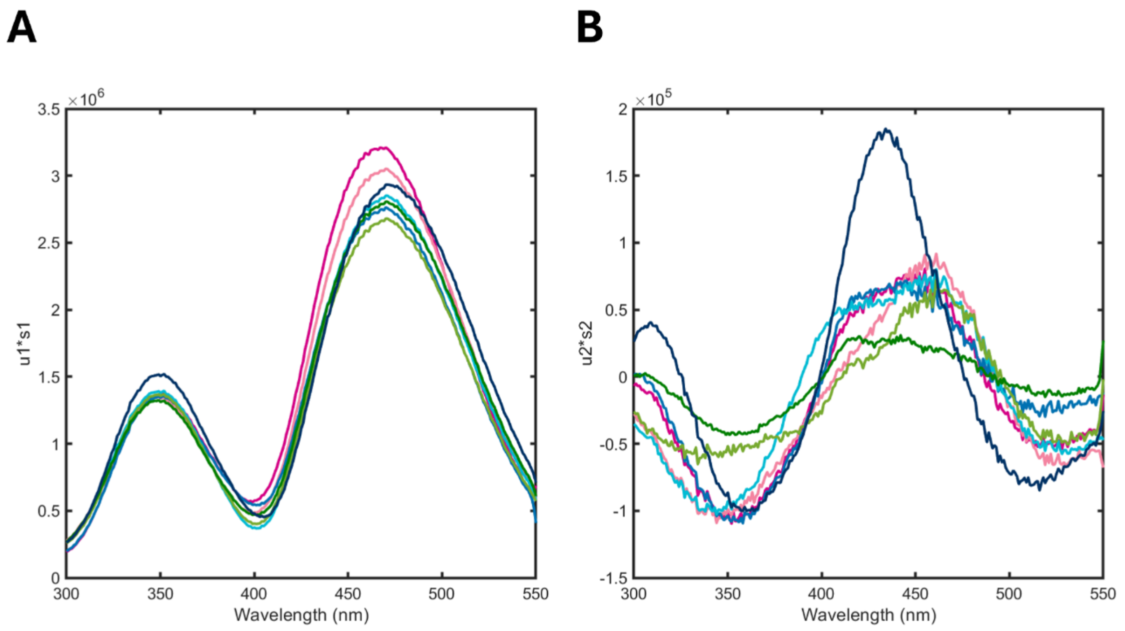

Figure S4. First (A) and second (B) eigenvectors (u) multiplied by the corresponding singular values (s) from SVD on each tested ligand with albuminodan: DPPC (dark green), POPG (dark blue), LPPG (light blue), LOPG (cyan) and LPPC (light green), oleic acid (magenta), and palmitic acid (light pink).

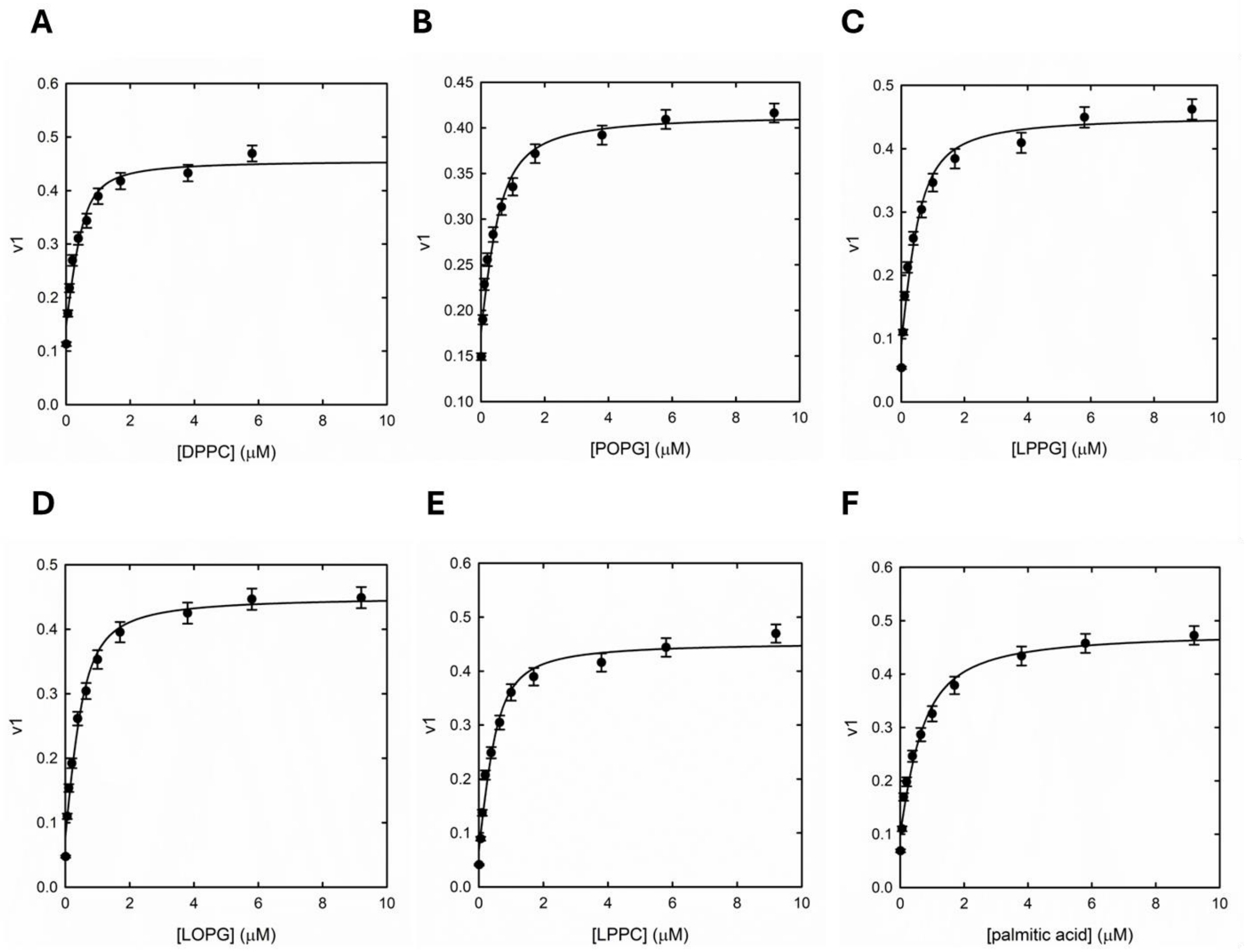

Figure S5. First component weight (eigenvalue) as a function of ligand concentration: DPPC (A), POPG (B) LPPG (C), LOPG (D), LPPC (E), palmitic acid (F) in presence of 500 nM albuminodan. The line through data points represents fitting to Equation 1. The error bars are obtained through jackknife resampling (leave-one-out variant).

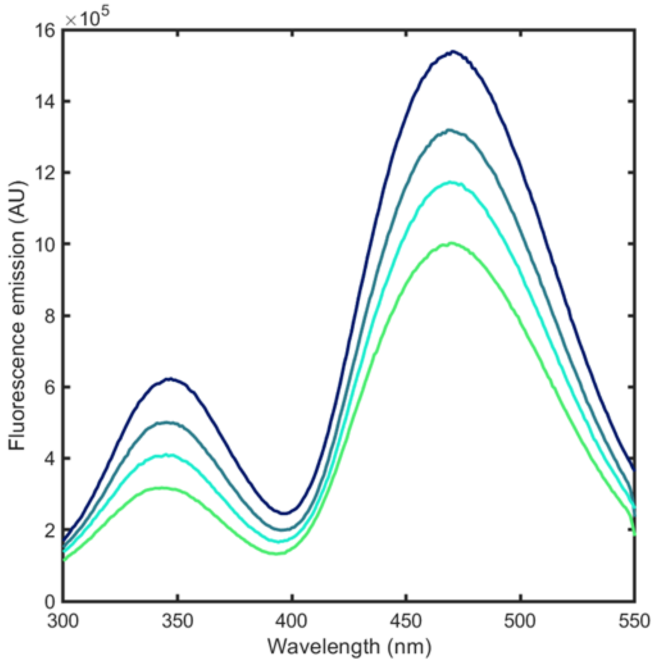

Figure S6. Fluorescence emission spectra upon excitation at 280 nm of albuminodan (dark blue) (69 μL) upon addition of 1 (dark cyan), 2 (cyan) and 3 (green) μL of a DPPC/POPG lipid-based formulation.
